# Variation of health-related quality of life assessed by caregivers and patients affected by severe childhood infections

**DOI:** 10.1186/1471-2431-13-122

**Published:** 2013-08-13

**Authors:** Wantanee Kulpeng, Vorasith Sornsrivichai, Virasakdi Chongsuvivatwong, Waranya Rattanavipapong, Pattara Leelahavarong, John Cairns, Yoel Lubell, Yot Teerawattananon

**Affiliations:** 1 Health Intervention and Technology Assessment Program (HITAP), Ministry of Public Health, Nonthaburi, Thailand; 2 Epidemiology Unit, Faculty of Medicine, Prince of Songkla University, Songkla, Thailand; 3 London School of Hygiene and Tropical Medicine, London, United Kingdom; 4 Mahidol Oxford Tropical Medicine Research Unit, Faculty of Tropical Medicine, Mahidol University, Bangkok, Thailand

**Keywords:** Infection, Chronic conditions, Child, Health-related quality of life, Utility, Proxy

## Abstract

**Background:**

The agreement between self-reported and proxy measures of health status in ill children is not well established. This study aimed to quantify the variation in health-related quality of life (HRQOL) derived from young patients and their carers using different instruments.

**Methods:**

A hospital-based cross-sectional survey was conducted between August 2010 and March 2011. Children with meningitis, bacteremia, pneumonia, acute otitis media, hearing loss, chronic lung disease, epilepsy, mild mental retardation, severe mental retardation, and mental retardation combined with epilepsy, aged between five to 14 years in seven tertiary hospitals were selected for participation in this study. The Health Utilities Index Mark 2 (HUI2), and Mark 3 (HUI3), and the EuroQoL Descriptive System (EQ-5D) and Visual Analogue Scale (EQ-VAS) were applied to both paediatric patients (self-assessment) and caregivers (proxy-assessment).

**Results:**

The EQ-5D scores were lowest for acute conditions such as meningitis, bacteremia, and pneumonia, whereas the HUI3 scores were lowest for most chronic conditions such as hearing loss and severe mental retardation. Comparing patient and proxy scores (*n* = 74), the EQ-5D exhibited high correlation (*r* = 0.77) while in the HUI2 and HUI3 patient and caregiver scores were moderately correlated (*r* = 0.58 and 0.67 respectively). The mean difference between self and proxy-assessment using the HUI2, HUI3, EQ-5D and EQ-VAS scores were 0.03, 0.05, -0.03 and -0.02, respectively. In hearing-impaired and chronic lung patients the self-rated HRQOL differed significantly from their caregivers.

**Conclusions:**

The use of caregivers as proxies for measuring HRQOL in young patients affected by pneumococcal infection and its sequelae should be employed with caution. Given the high correlation between instruments, each of the HRQOL instruments appears acceptable apart from the EQ-VAS which exhibited low correlation with the others.

## Background

Measuring health-related quality of life (HRQOL) is increasingly used to quantify the effect of a health condition on an individual’s life, and to assess the impact of health care interventions. Economic evaluations measure HRQOL in terms of utility, which can be subsequently incorporated along with changes in life expectancy in the calculation of Quality-Adjusted Life Years (QALYs) to compare health outcomes across health interventions in different diseases and disabilities to inform resource allocation. Utility scales usually range from 0 to 1, where full health is assumed to have the value 1 and death the value 0. Some HRQOL measures such as the Health Utilities Index Mark 2 (HUI2), and Mark 3 (HUI3), and EuroQoL Descriptive System (EQ-5D) allow negative scores that express health states considered worse than death.

There are difficulties and limitations in assessing HRQOL in young children. Firstly, children’s growth and development changes rapidly, which may affect the baseline measure of particular health dimensions such as self-care, usual activity or communication ability [1,2]. At present, there are no standard instruments for measuring health status in this population. While the HUIs and EQ-5D, generic health status instruments are recognised as valid and reliable for eliciting health status in adults and children aged over four years (for the HUIs and through proxy-assessment) or 14 years (for the EQ-5D) [3-7], and are widely used in cost-utility analysis (CUA) [1,8], their application for younger age-groups is still controversial [1]. Furthermore, HRQOL obtained using different instruments can differ substantially even when measured in the same person [9,10] a phenomenon that is particularly evident in young children. While some variation in HRQOL scores obtained from different instruments is inevitable, these can be tested in target populations in order to explore the extent of variation between them. Instruments that provide widely differing outcomes might then be considered less appropriate for use in these populations.

A second challenge to the use of HRQOL instruments with young children, is that these should ideally be completed by the target population, posing substantial challenges in very young responders. A review found that only 2% of studies where children were the primary beneficiaries of the intervention estimated HRQOL scores directly from this age-group [1]. This is expected given the greater difficulties children might face in accurately describing their health condition during and after illness episodes. In addition, some of the questions might be too complex for young children to answer. As a result, proxy-assessment, where children’s health status is obtained through their caregivers, physicians, or adult patients with similar health conditions, is applied [11-14]. However, self- and proxy-assessed HRQOL scores may vary, even when using the same tools [12,13].

Based on this review, two potential sources of variation are present when assessing HRQOL in young children: 1) variation due to the choice of instrument; 2) variation between the measures obtained from patients directly as opposed to their carers. The agreement between self-reported and proxy measures of health status in ill children is not well established and there are no clear guidelines as to whether this is acceptable practice [12,13,15,16]. Where the use of a proxy is not appropriate, better guidance is needed on the most appropriate tools for health status measurement in young children.

This study explores the use of instruments for HRQOL measurement in young children affected by infectious diseases in Thailand, and is a part of a CUA of 10- and 13-valent pneumococcal conjugate vaccines. Assessment using various HRQOL instruments by the caregivers and affected children (who are able to rate their health status) can provide the necessary data to address the above knowledge-gap.

The specific objectives of this study are to 1) quantify the variation in scores derived from young patients and their carers using different HRQOL instruments in different health conditions; 2) provide recommendations as to whether it is appropriate to measure HRQOL of paediatric patients using their caregivers’ assessments; 3) where proxy assessment is not appropriate, identify which instrument is most suitable for use in very young children.

## Methods

### Study design and sample

The health conditions to be assessed in this study were selected by a consortium of experts in paediatric infectious disease, paediatric neurology, epidemiology, vaccinology, and health economics. The list of conditions aimed to include the most common severe pneumococcal infections and their sequelae that are likely to have the highest impact on HRQOL. The final list included: 1) meningitis 2) bacteremia, 3) pneumonia, 4) acute otitis media (AOM), 5) hearing loss, 6) chronic lung disease, 7) epilepsy, 8) mild mental retardation (MMR), 9) severe mental retardation (SMR), and 10) mental retardation combined with epilepsy (MR + epilepsy).

We conducted a hospital-based cross-sectional survey from August 2010 to March 2011 in seven public tertiary hospitals in different parts of Thailand. The hospitals were selected based on having a high number of bacterial meningitis cases which was a relatively rare condition but one with a high burden of disease. This study was approved by the ethics committee of Queen Sirikit National Institute of Child Health, Nopparat Rajathanee Hospital, Maharat Nakhon Ratchasima Hospital, Udonthani Hospital, Chiangrai Regional Hospital, Hatyai Hospital, and Faculty of Medicine, Prince of Songkla University. We calculated the sample size based on a attempt to detect a mean difference of 0.05 of the maximum of various scales for HRQOL for patient-caregiver pairs with and an estimated standard deviation (SD) of paired response difference of 0.03 [17], a power of 80% with a significance level of 0.05, at least six pairs were required for each health condition.

Health personnel from the study sites helped in the identification of eligible patients and their caregivers. Pneumococcal bacteremia, pneumococcal pneumonia and bacterial meningitis cases were identified in the paediatric wards. The case definition for these cases conformed with the clinical criteria defined by the Case Definitions for Infectious Conditions in Thailand [18] or the International Classification of Diseases and Related Health Problems (10^th^ edition). AOM, hearing loss, chronic lung disease, epilepsy, MMR, SMR and MR + epilepsy cases were identified in the paediatric clinics. Relevant outpatient cases were classified into each health condition according to physicians’ diagnosis, regardless of diagnostic method. We selected all cases who met the criteria during the data collection period.

Patients aged between five and 14 years in the selected hospitals who were diagnosed with any single one of the selected health conditions were enrolled. All school age patients aged 7 years and above who were able to communicate were invited along with their caregivers to directly participate in the study. For patients aged under seven years and for patients who refused or were unable to answer a series of questions, such as those with mental retardation (MR), only caregivers were invited to participate. Caregivers were excluded if they were unable to answer the questions or unwilling to participate in the study.

Consent was sought from a parent or guardian of the identified patients prior to interviews and reviews of paediatric medical records. The participants were interviewed by interviewers using the Thai version of HUIs and EQ-5D questionnaires. Although these instruments can routinely be completed independently by patients, in the study both patients and caregivers were interviewed face-to-face by well-trained interviewers reading out the structured questionnaires and themselves completing the forms.

### Study instruments

The HUI2, HUI3 and EQ-5D were selected as they have been widely used in HRQOL measurement in children and Thai versions have already been validated and approved by the Health Utilities Inc and the EuroQol group, respectively. In addition, responses can be converted into utility scores. The EQ-VAS is an integral component of the EQ-5D questionnaire; however, we examine it here independently of the primary descriptive system. From our literature review, all of these scales were reported to have minimal problem of floor and ceiling effects with the exception of an important ceiling effect in the case of EQ-5D [19-23].

The EQ-5D includes five dimensions (mobility, self-care, usual activities, pain/discomfort, and anxiety/depression) with three ordered levels of severity for each dimension. The self-administered version of EQ-5D is considered suitable for people aged 14 years and above. An EQ-5D youth (EQ-5D-Y) version for children aged between seven to 12 years has been developed but has not been adapted to the Thai context. The EQ-VAS is a standardised extension to the EQ-5D descriptive system. It is a rating scale with a vertical 20 cm Visual Analogue Scale (VAS) with the end points labelled best imaginable health state at the top and worst imaginable health state at the bottom having numeric values of 100 and 0, respectively. The standard version was used for all subjects.

The HUI2 comprises seven dimensions (sensation, mobility, emotion, cognition, self-care, and pain and fertility) with four or five ordered levels of severity for each dimension. The HUI3 was developed to address concerns surrounding certain definitions in the HUI2 [24], and is comprised of eight dimensions (vision, hearing, speech, cognition, pain, emotion, ambulation, and dexterity) with five or six ordered levels of severity for each dimension. Of the seven dimensions in HUI2, the fertility dimension was excluded, whereas the sensation dimension was split into vision, hearing and speech. We used the validated Thai version ‘HUI23′ [25], which includes all 41 questions that comprise HUI2 (37 questions) and HUI3 (33 questions), and from which each instrument can be used by selecting the relevant components. The HUIs have been considered suitable for people aged five years and above through proxy-assessment.

### Data analysis

A Thai algorithm was used to calculate the EQ-5D scores [26] but a Canadian scoring function of HUIs was used for HUI23 due to the lack of local data [27]. The correlation between scores from different instruments was calculated for patients and for caregivers, and the correlation between scores from patients and caregivers was calculated for different instruments. To determine whether there were systematic differences in scores between instruments, we calculated for each health condition and overall HRQOL the mean score and its 95% confidence interval (CI) using each of the HRQOL instruments in both paediatric patients where possible, and in their caregivers. ANOVA was used to analyze the source of variability of the scores. Differences in scores between caregivers and patients were tested using paired t-tests for the 28 condition-instrument combinations. All statistical analyses were carried out in the open source R software package [28].

## Results

In total 173 cases were identified. None of the caregivers refused to participate giving a 100% response rate. The number of respondents by health conditions is shown in Table 1. A total of 74 paediatric patient-caregiver complete sets participated in this study. Additionally, 99 caregivers participated with a corresponding patient that was either too sick (all cases of MR and the majority of meningitis, pneumonia and AOM, *n* = 53) or were too young (aged less than 7 years, *n* = 46) to complete the questionnaire. The overall mean patient age was 10 (SD = 3). Males accounted for 62%. Among 173 caregivers, the mean age was 40 years (SD = 11) and males accounted for only 13%. The duration for completing the HUI23 was approximately eight minutes in both patients and caregivers, significantly longer than for the EQ-5D + VAS which took approximately three minutes.

**Table 1:** Number of assessors by health conditions

**Health conditions**	**Total 173 cases**	
	**Assessed by caregivers and paediatric patients**	**Assessed by caregivers alone**
**Acute**		
Meningitis	7	12
Bacteremia	9	7
Pneumonia	8	16
AOM	7	11
**Sequalae**		
Hearing loss	15	7
Chronic lung disease	12	4
Epilepsy	16	4
MMR	0	8
SMR	0	11
MR + epilepsy	0	18
**Total**	**74**	**99**

Table 2 shows correlation coefficients among different instruments in the same subjects (both the patient and caregiver) and between the same patient-caregiver pair using the same instrument (highlighted in the bold). Most values indicated relatively high or moderate correlation except the correlation coefficients between the EQ-VAS and HUIs, both within the same person and between patient and caregiver in the same pair.

**Table 2:** **Matrix of scores obtained in paediatric patients and caregivers using 4 HRQOL instruments (*N*** **= 74)**

	**Scores from caregiver**			**Scores from patient**			
	**HUI3**	**EQ-5D**	**EQ-VAS**	**HUI2**	**HUI3**	**EQ-5D**	**EQ-VAS**
**Scores from caregiver**							
HUI2	0.84	0.63	0.43	**0.58**	0.57	0.56	0.20^a^
HUI3		0.69	0.50	0.58	**0.67**	0.59	0.24
EQ-5D			0.55	0.40	0.44	**0.77**	0.49
EQ-VAS				0.11^a^	0.20^a^	0.40	**0.50**
**Scores from patient**							
HUI3				0.89			
EQ-5D				0.59	0.58		
EQ-VAS				0.11^a^	0.16^a^	0.37	

Values in bold correspond to the correlation coefficients of the scores between the patients and the caregivers using the same instruments. ^a^The correlation is found to be non-significant (*P* > 0.05).

**Figure 1:**
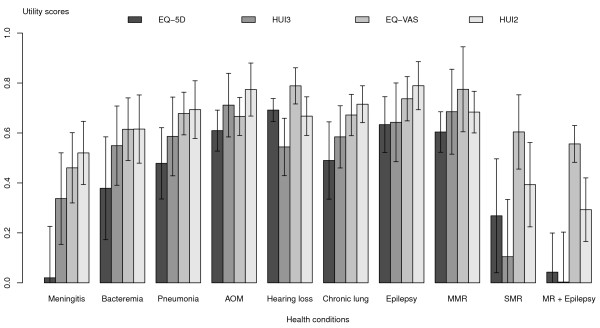
Mean scores and 95% CI obtained in caregivers using 4 HRQOL instruments (proxy-assessment).

**Figure 2:**
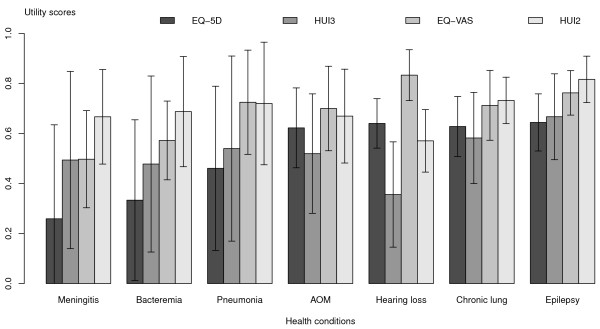
Mean scores and 95% CI obtained in paediatric patients using 4 HRQOL instruments (self-assessment).

The HRQOL scores obtained from all caregivers are shown in Figure 1. The EQ-5D scores are the lowest for seven of 10 health conditions i.e., meningitis, bacteremia, pneumonia, AOM, chronic lung disease, epilepsy and MMR, whereas the HUI3 gave the lowest scores for three health conditions i.e., hearing loss, SMR and MR + epilepsy. The HRQOL scored by paediatric patients themselves are shown in Figure 2. Similarly, the EQ-5D scores were lowest among four of the seven health conditions in which patients could respond i.e., meningitis, bacteremia, pneumonia and epilepsy. Likewise, the HUI3 scores were lowest in the remaining three conditions. We ran a factor analysis for the mean of each measure on each health condition. Two factors were identified in both caregiver and patient data sets. In both groups, the first factor included meningitis, bacteremia, pneumonia, chronic lung disease and epilepsy. The second factor had less consistent components. The total variances of these means explained by the two factors were 94% in caregivers and 98% in patients.

Table 3 illustrates the source of variation in HRQOL scores in the two data sets. Using data from the complete sets, variation within the same patient-caregiver pair was small and not significant (*P* = 0.59). Variation contributed by difference in the health conditions and instruments were highly significant in both data sets. When accounting for interaction between the health conditions and instruments, the *P* was small indicating that both health conditions and instruments were not acting independently from each other.

**Table 3:** Comparison of mean scores by sources

	**Df**	**SS**	**MS**	**F**	**P-value**
**From caregivers and patients complete sets**					
Assessor	1	0.02	0.02	0.28	0.59
Health condition	6	4.02	0.67	9.57	<0.001
Instrument	3	3.85	1.28	18.31	<0.001
Health condition : instrument	18	2.99	0.17	2.49	<0.001
Residuals	581	40.66	0.07		
**From sets with caregivers only**					
Health condition	9	15.29	1.70	27.38	<0.001
Instrument	3	3.51	1.05	16.92	<0.001
Health condition : instrument	27	3.12	0.12	1.86	<0.01
Residuals	652	40.47	0.06		

Table 4 shows the breakdown of differences within caregiver-patient sets by health condition and instruments. HRQOL reported by paediatric patients were slightly and non-significantly higher than those reported by caregivers. The only significant difference detected within the pairs was for hearing loss using HUI3 and chronic lung disease using EQ-5D.

**Table 4:** Mean of difference of scores between caregivers and paediatric patients

			**Mean of difference**			
**Health conditions**		***N***	**HUI2**	**HUI3**	**EQ-5D**	**EQ-VAS**
Meningitis		7	-0.07	-0.07	-0.27	0.05
	(95%CI)		(-0.17 to 0.03)	(-0.21 to 0.07)	(-0.58 to 0.05)	(-0.09 to 0.20)
Bacteremia		9	0.05	0.13	0.08	0.01
	(95%CI)		(-0.15 to 0.24)	(-0.18 to 0.44)	(-0.13 to 0.29)	(-0.19 to 0.21)
Pneumonia		8	-0.08	-0.02	-0.05	-0.05
	(95%CI)		(-0.22 to 0.07)	(-0.13 to 0.10)	(-0.15 to 0.05)	(-0.28 to 0.17)
AOM		7	0.05	0.07	-0.08	-0.06
	(95%CI)		(-0.09 to 0.19)	(-0.09 to 0.24)	(-0.20 to 0.04)	(-0.23 to 0.11)
Hearing loss		15	0.14	0.24	0.08	0.01
	(95%CI)		(-0.01 to 0.28)	(0.03 to 0.46)^a^	(-0.03 to 0.19)	(-0.12 to 0.14)
Chronic lung		12	0.00	0.03	-0.11	-0.03
	(95%CI)		(-0.11 to 0.12)	(-0.09 to 0.14)	(-0.22 to -0.00)^a^	(-0.16 to 0.09)
Epilepsy		16	-0.01	0.00	-0.00	-0.02
	(95%CI)		(-0.08 to 0.07)	(-0.11 to 0.12)	(-0.06 to 0.06)	(-0.10 to 0.06)
**Overall**		**74**	**0.03**	**0.05**	**-0.03**	**-0.02**
	**(95%CI)**		**(-0.02 to 0.07)**	**(-0.00 to 0.11)**	**(-0.07 to 0.02)**	**(-0.06 to 0.02)**

^a^Statistically significant (*P* < 0.05) different utility score for caregivers compared with patients.

## Discussion

This is the first study considering methodological aspects of children’s HRQOL instruments in the Thai context and results of this study can be useful for guiding future economic evaluations or outcome studies in this and other settings. In this analysis, we address two major methodological issues concerning the use of caregivers as proxies for children’s HRQOL measures, and the use of different HRQOL instruments across health conditions in young patients.

### The variation in HRQOL derived from patients compared to their caregivers

We observed disparity in HRQOL derived from young patients and caregivers using all instruments, and the mean of differences exceeded 0.03, a difference that has been considered to be clinically significant by previous investigators [7,9,29]. Likewise, the data in Table 4 suggest that the difference between patients and caregivers was in the majority of health conditions of a magnitude which would be regarded as clinically meaningful though not statistically significant (except for hearing loss and chronic lung disease using particular instruments). The largest gap was found in hearing loss. Health conditions relating to sensory impairment such as hearing loss might be more challenging in proxy-assessment than objective measures such as mobility.

The variation in HRQOL scores derived from patients compared to their caregivers was also associated with HRQOL instruments. The HUIs and EQ-5D scores had good correlation within patient-caregiver pairs, a finding that is also compatible with other studies [14,30,31]. The degree of caregiver-patient correlation in the HUI3 was higher than in the HUI2 in our study. The EQ-VAS in both patients and caregivers had the lowest correlation with other measures. This may be because the EQ-VAS involves a different task (valuation of health state) whereas for the other three measures the respondents were asked to describe their own or the child’s health state.

### The use of different HRQOL instruments across health conditions in young patients

As would be expected, all instruments offered different HRQOL scores for the same health condition. For both self- and proxy-assessment, the EQ-VAS and HUI2 gave the highest scores whereas the EQ-5D and HUI3 tended to provide the lowest. The EQ-5D yielded the lowest HRQOL scores compared to other instruments in acute diseases, whereas the HUI3 provided the lowest score in most of chronic conditions. These findings are consistent with two other studies [10,32]. Our study, however, found that for epilepsy the HRQOL score was the lowest using the EQ-5D, as opposed to HUI3 in another study [10]. It is noteworthy that the Thai algorithm used for EQ-5D was derived from the Time Trade-Off (TTO) technique, whereas HUI scoring function was obtained from the Standard Gamble (SG) technique and VAS. This difference might influence the results because previous studies indicated that TTO produced lower utility scores than SG in Asian and other population groups [33-36]. Moreover, the absence of a Thai specific scoring function for HUIs could have affected the results as people in different countries are likely to have different health state preferences [32].

The EQ-5D in particular may not be sufficiently sensitive for measuring HRQOL in patients with sensory impairment as it does not include a sensory dimension [32,37-39]. SG and TTO have been used to measure utility directly in hearing impaired persons [40]. The SG and TTO, however, are time-consuming and conceptually challenging. Furthermore, the HUI3 has proven to be valid and acceptable for measuring HRQOL in hearing impaired populations [10,32,37-39,41]. For health conditions associated with sensory impairment, therefore, self-reported assessment of health status using the HUI3 is the optimal choice. The EQ-VAS score obtained from patients and caregivers is similar, yet correlation between scores rated by this and other instruments was low. Furthermore, given the general difficulties in using the EQ-VAS in people who may not understand its quantitative properties [33,42,43], it may not be appropriate for very young patients. This was supported by a prior study showing that 13% of adult patients found it difficult to use [43].

In addition, the degree of correlation between instruments is used to examine their agreement (convergent validity). The HUIs and EQ-5D scores had a moderate to high correlation within the same subject, confirming findings from previous studies [44-46]. The HUI2 and HUI3 had very high correlation; this is mainly because there is much duplication in these tools (30 of 40 questions in HUI23 are identical). The HUI3 is claimed to be superior to the HUI2 as it was developed to improve structural independence so that each domain would yield specific information [24,47].

### Study limitations

In addition to the limitation of incomplete pairs of patient-caregiver sets, another methodological concern is the fact that subjects were recruited at tertiary hospitals where patients are likely to be in an acute phase of their illness and the impact on certain HRQOL dimensions such as mobility may not be readily apparent. We argue that this did not introduce a substantial bias since patients in most of our pre-specified conditions are usually hospitalized. The shortcoming may be more serious in health states associated with chronic disability as patient and proxy assessment of their HRQOL once back home may be different from when they are hospitalized [48]. Lastly, although this study selected patients with a single condition, there may have been co-morbidities that were undiagnosed during data collection that may have influenced HRQOL scores.

## Conclusions

Our data imply that use of caregivers as proxies for measuring HRQOL in young patients affected by pneumococcal infection and its sequelae should be employed with caution. Given the high correlation between instruments, each of the HRQOL instruments appears acceptable apart from the EQ-VAS which exhibited low correlation with the others. For conditions associated with sensory impairment we would recommend the use of HUI3 due to its explicit inclusion of this dimension.

## Abbreviations

HRQOL: Health-related quality of life; QALYs: Quality-adjusted life years; HUI2: Health Utilities Index Mark 2; HUI3: Health Utilities Index Mark 3; EQ-5D: EuroQoL Descriptive System; VAS: Visual Analogue Scale; TTO: Time Trade-Off; SG: Standard Gamble; CUA: Cost-utility analysis; AOM: Acute otitis media; MR: Mental retardation; MMR: Mild mental retardation; SMR: Severe mental retardation; MR + epilepsy: Mental retardation combined with epilepsy; CI: Confidence interval; SD: Standard deviation; r: Correlation coefficient.

## Competing interests

The authors declare that they have no completing interests.

## Authors’ contributions

WK, VS, VC, JC and YT contributed to conception and study design. WK and VC analyzed and interpreted data. WK drafted the manuscript. All authors contributed to the analysis and revised the manuscript. All authors read and approved the final manuscript.

## Pre-publication history

The pre-publication history for this paper can be accessed here:

http://www.biomedcentral.com/1471-2431/13/122/prepub
